# Metacognition of ChatGPT in confidence judgements

**DOI:** 10.3389/frai.2026.1694192

**Published:** 2026-03-27

**Authors:** Shun Yoshizawa, Ayako Onzo, Shinichi Nozawa, Tsugumi Takano, Tetsuo Ishikawa, Ken Mogi

**Affiliations:** 1 Department of Physics, School of Science, Tokai University, Hiratsuka, Kanagawa, Japan; 2 Sony Computer Science Laboratories, Tokyo, Japan; 3 UT-Lab Institute, Tokyo, Japan; 4 Collective Intelligence Research Laboratory, Graduate School of Arts and Sciences, The University of Tokyo, Tokyo, Japan; 5 Department of General Systems Studies, Graduate School of Arts and Sciences, The University of Tokyo, Tokyo, Japan; 6 Department of Extended Intelligence for Medicine, The Ishii-Ishibashi Laboratory, Keio University School of Medicine, Tokyo, Japan; 7 Division of Applied Mathematical Science, Center for Interdisciplinary Theoretical and Mathematical Sciences (iTHEMS), RIKEN, Yokohama, Kanagawa, Japan

**Keywords:** AI alignment, confidence judgements, hallucination, large language model, metacognition

## Abstract

Recent advances in Large Language Models (LLMs) have raised critical concerns regarding AI alignment and safety, particularly with respect to the reliability of their outputs. In humans, metacognition plays a key role in making cognition robust and adaptive. LLMs frequently express high confidence in their responses, raising the question of whether such confidence reflects human-like metacognitive capability. In this study, we systematically compared humans and GPT-4 across multiple task formats to examine how confidence relates to performance. GPT-4 consistently outperformed humans in task accuracy. This advantage was not accompanied by human-like confidence behavior: Human confidence closely tracked variations in accuracy, while GPT-4 was not. Humans adjusted their confidence more sensitively to changes in accuracy, whereas GPT-4 showed a shallow confidence–accuracy mapping. Humans exhibited higher and more stable metacognitive sensitivity and efficiency, while GPT-4 showed condition-specific variability. These findings reveal a dissociation between task-level performance and metacognitive behavior in GPT-4, suggesting that its confidence reflects structural properties of its outputs rather than genuine internal uncertainty monitoring. Taken together, these findings suggest that GPT-4 lacks robust metacognitive abilities compared to humans, or at least that its metacognitive processes differ significantly from those of humans.

## 1 Introduction

Various AI systems, especially generative AIs, have made significant progress in recent years. One of the LLMs, the Generative Pre-Trained Transformer 4 (GPT-4, OpenAI et al., 2024a) showed never-before-seen performances including the emulation of human cognition (Frank, 2023), inference and reasoning (Bubeck et al., 2023; Webb et al., 2023), and theory of mind (ToM; Kosinski, 2024; Strachan et al., 2024). Making comparisons between humans and AI is important for assessing AI ability (Turing, 1950; Jones and Bergen, 2025), enhancing AI alignment (Yudkowsky, 2015), AI safety (Falco et al., 2021; Costello et al., 2024; Salvi et al., 2025), and human-AI collective intelligence (Vaccaro et al., 2024; Heyman et al., 2024; Malone and Bernstein, 2015). Sartori and Orrù (2023) argued that the size and complexity of a typical LLM makes it impossible to understand its behavior by looking at the model’s architecture and training corpus. Instead, they argue that it is important to explore an LLM empirically, as psychologists do.

LLMs have been studied as a cognitive model (Frank, 2023; Binz and Schulz, 2023; Shiffrin and Mitchell, 2023) in comparison with humans. In a false belief task related to ToM (Baron-Cohen, 1995), ChatGPT showed performance partially comparable to humans (Kosinski, 2024; Strachan et al., 2024). The cognitive capability of LLMs has been analyzed and evaluated from contexts of computational psychiatry (Coda-Forno et al., 2024) and Big Five personality traits (Serapio-García et al., 2025).

In humans, metacognition has been studied by measuring confidence in responses explicitly (Kepecs and Mainen, 2012; Hart, 1965), and in relation to the feeling-of-knowing (FOK; Koriat, 1993). Metacognition has also been studied in AI (Fleming, 2021). LLMs are known to hallucinate (Huang et al., 2025), possibly due to the lack of metacognition. The failure to judge the correctness of responses is regarded as one of the major challenges to be overcome in order to constitute a reliable AI (Yampolskiy, 2018). Anthropic researchers have introduced a probability function called P(IK), the probability that “I know” the answer to a question, in LLMs in an effort to reduce hallucination (Kadavath et al., 2022), purportedly reproducing FOK in humans. Hallucination in LLMs might correlate with the lack of processes related to metacognition. Does an LLM have a tendency to exhibit less confidence in its outputs as tasks become more uncertain and difficult?

Fleming and Lau (2014) proposed classifying metacognitive ability into three related concepts: metacognitive sensitivity, metacognitive efficiency, and metacognitive bias. Metacognitive sensitivity is the correlation between accuracy and confidence in a given task of a participant. The level of metacognitive sensitivity in a participant given a certain level of task performance is metacognitive efficiency. Metacognitive bias is the difference in a participant’s confidence level in the absence of task performance change, where, for example, people with shy personalities are always less confident (Fleming and Lau, 2014). It is known that metacognitive sensitivity is influenced by metacognitive bias (Fleming and Lau, 2014; Nelson, 1984). Another experimental paradigm for measuring metacognition is the Uncertainty Response (UR) paradigm (Smith and Washburn, 2005). The UR is an implicit indicator of metacognition that the behavior of the participant changes when the uncertainty of the task changes. It has been used mainly for animal subjects that cannot report verbally, and made it possible to compare metacognitive abilities between humans and animals (Kepecs and Mainen, 2012; Shields et al., 2005). The folded X-pattern is one of the statistical signature analyses used to evaluate confidence in decision-making (Kepecs and Mainen, 2012; Shields et al., 2005; Fleming, 2024; Rausch and Zehetleitner, 2019). It describes a specific relationship between confidence, accuracy, and stimulus discriminability. The critical aspects of the folded X-pattern are the increase of confidence in correct choices as the discriminability of the stimulus increases, and the decrease in confidence in incorrect choices as the discriminability of the stimulus increases.

Cash et al. (2025) systematically evaluated LLMs’ metacognitive abilities across various tasks in which the models (ChatGPT (GPT-4o), Bard/Gemini, Claude Sonnet, and Haiku) were asked to report their confidence in their own performances. They included two types of confidence ratings: one collected before task execution (prospective confidence) and another collected afterwards (retrospective confidence). A decrease in post-task confidence despite initially high pre-task confidence—particularly when actual performance was poor—would suggest that the LLM is capable of updating its metacognitive evaluation through an interaction.

In LLMs, uncertainty is commonly divided into two classes: aleatoric uncertainty, which reflects irreducible variability inherent in the data, and epistemic uncertainty, which stems from the model’s limited knowledge or ignorance (Hou et al., 2024). Tasks in Cash et al. (2025) included those involving aleatoric uncertainty and those involving epistemic uncertainty. One of the aleatoric-uncertainty tasks used was Oscar prediction (humans and LLMs predict the winner of nine Oscar categories), and one of the epistemic-uncertainty tasks was answering trivia questions. They also used type-2 AUROC to assess relative metacognitive accuracy, thereby enhancing methodological rigor and reproducibility.

From the analysis of these tasks, Cash et al. (2025) found that LLMs showed metacognitive accuracy equivalent to or slightly higher than that of humans and tended to be overconfident. In particular, ChatGPT was overconfident in trivia questions, whereas humans were underconfident.

Griot et al. (2025) likewise reported that various LLMs, including Meta-Llama-3-70B and Meta-Llama-3-8B (Grattafiori et al., 2024), tend to exhibit overall overconfidence. Using multiple-choice medical-knowledge questions, they found that GPT-4o (OpenAI et al., 2024b) and GPT-3.5-turbo often gave confident answers even when no correct option was present, though GPT-4o showed relatively better calibration. Based on such findings, Griot et al. (2025) argued that LLMs display a significant metacognitive deficiency; however, they did not compare LLMs with humans. In sum, it remains to be examined how LLMs’ metacognitive abilities compare with those of humans.

Based on the UR paradigm, we designed three categories of answering about general knowledge for human participants and LLMs: two alternative choices (2C), four alternative choices (4C), and open-ended response (OP), with different uncertainty levels (chance levels of 0.5, 0.25 and 0, respectively). The chance level of 0 for OP is not in the strict mathematical sense, but for all practical purposes. We compared how accuracy and confidence varied between LLMs and humans across the open-ended, four-choice, and two-choice conditions. We presented the questions in the order of open-ended (OP), two-choice (2C), and four-choice (4C) conditions to LLMs and humans.

The task was designed based on Smith’s Uncertainty Response (Smith and Washburn, 2005) paradigm. The UR is an implicit metacognition measure that uses changes in the participant’s behavior when the uncertainty of the task is changed as the basis for metacognition. This paradigm is also useful for animals that cannot perform verbal reports. The behavior of LLMs would be able to be assessed by such tasks in a continuous spectrum with animals. More details for these conditions are given later in Materials and Methods and Results sections. To remove the influence of bias on humans and GPT-4, we conducted four types of analysis, i.e., the Type-2 Receiver Operating Characteristic (ROC) (Fleming and Lau, 2014), meta- J , and meta- $J_{2}^{r}$ (Dayan, 2023), and the folded X-pattern analysis. Meta- J and meta- $J_{2}^{r}$ are model-free (Fleming, 2024) and bias-free measures.

In the Type-2 ROC analysis, a larger Area Under the ROC Curve (AUROC) indicates a higher metacognitive sensitivity (Fleming and Lau, 2014). Meta- J is the mutual information (Cover and Thomas, 2006) between the accuracy (correct/incorrect) and the confidence level of a given task. Mutual information represents the correlation between two random variables. Meta- $J_{2}^{r}$ is the value of meta- J normalized by the upper bound of meta- J (Shannon entropy of accuracy (correct/incorrect)). The meta- J is the measure of metacognitive sensitivity, and meta- $J_{2}^{r}$ is the measure of metacognitive efficiency. We also evaluated the folded X-pattern by plotting the confidence in humans and GPT-4 correct and incorrect choices with respect to discriminability (correct rate).

We preliminarily evaluated GPT-4’s metacognition using confidence-judgement tasks and compared it with human performance (Yoshizawa et al., 2024). We further calculated type-2 AUROC and meta- J , as well as the folded X-pattern—established indices of metacognitive analysis—to compare the metacognitive capabilities of humans and GPT-4.

We analyzed the statistical relationships between accuracy (correct/incorrect) and confidence level (0–100%) to compare the metacognitive abilities of GPT-4 (*n* = 87) and humans (*n* = 87) through confidence judgements both explicitly and implicitly, under the default assumption that confidence level and correct rate would positively correlate in human participants, while GPT-4 would fail to exhibit such a correlation. We hypothesized that there would also be differences in metacognitive sensitivity between humans and GPT-4 when analyzing Type-2 AUROC, meta- J , and the folded X-pattern.

## 2 Materials and methods

Eighty-seven Japanese-speaking participants (37 females, 50 males, and 2 reporting other genders; mean age 46.3 ± 13.0 years) were recruited through the X (formerly Twitter) accounts of authors SY and KM, and answered questions through Google Forms. GPT-4 gave responses to the questions and corresponding confidence levels via Japanese prompts, in the same manner as the tasks administered to human participants. For both human participants (*n* = 87) and GPT-4 (87 independent runs), we calculated participant-level mean task accuracy (correct rate) and confidence scores. Participants were asked 60 general knowledge questions about Japan (geography, culture, nature & history) in 3 categories (2 alternative choice (2C), 4 alternative choice (4C) and open-ended (OP) questions), compiled by one of the authors (SY) and checked by other authors for appropriateness and accuracy. There were 20 questions in each category. Human participants were asked questions in the order OP, 2C, and 4C. 2C questions included: Is Lake Biwa the largest lake in Japan in terms of area, YES/NO? 4C questions included: Which Lake has the largest area in Japan? Lake Biwa/Lake Saroma/Kasumigaura/Nakaumi. OP questions included: What is the largest lake in Japan? After each response, participants reported a confidence level between 0 and 100%.

The same set of questions were asked of the GPT-4 87 times (to match the human sample size (*n* = 87)) using the API, in order to level out variabilities in responses within the constraints of available resource limits, and the average correct rates were calculated. The temperature parameter of GPT-4 was set to the default. For all API calls, the model parameter was set to “gpt-4,” and the maximum number of generated tokens was limited to 300. In the context of using GPT-4 through an API, the conditions defined within a task are mutually independent, and their ordering has no effect on the results. The code used for GPT-4 data generation is publicly available (see Data Availability Statement).

The questions were asked in Japanese to both humans and the GPT-4. The full list of questions is available as described in Data availability. The experiment was conducted between September and October 2023 for humans, and in December 2025 for the GPT-4. Possible variabilities in the responses of GPT-4, if any, were constrained by this time period.

**Figure 1:**
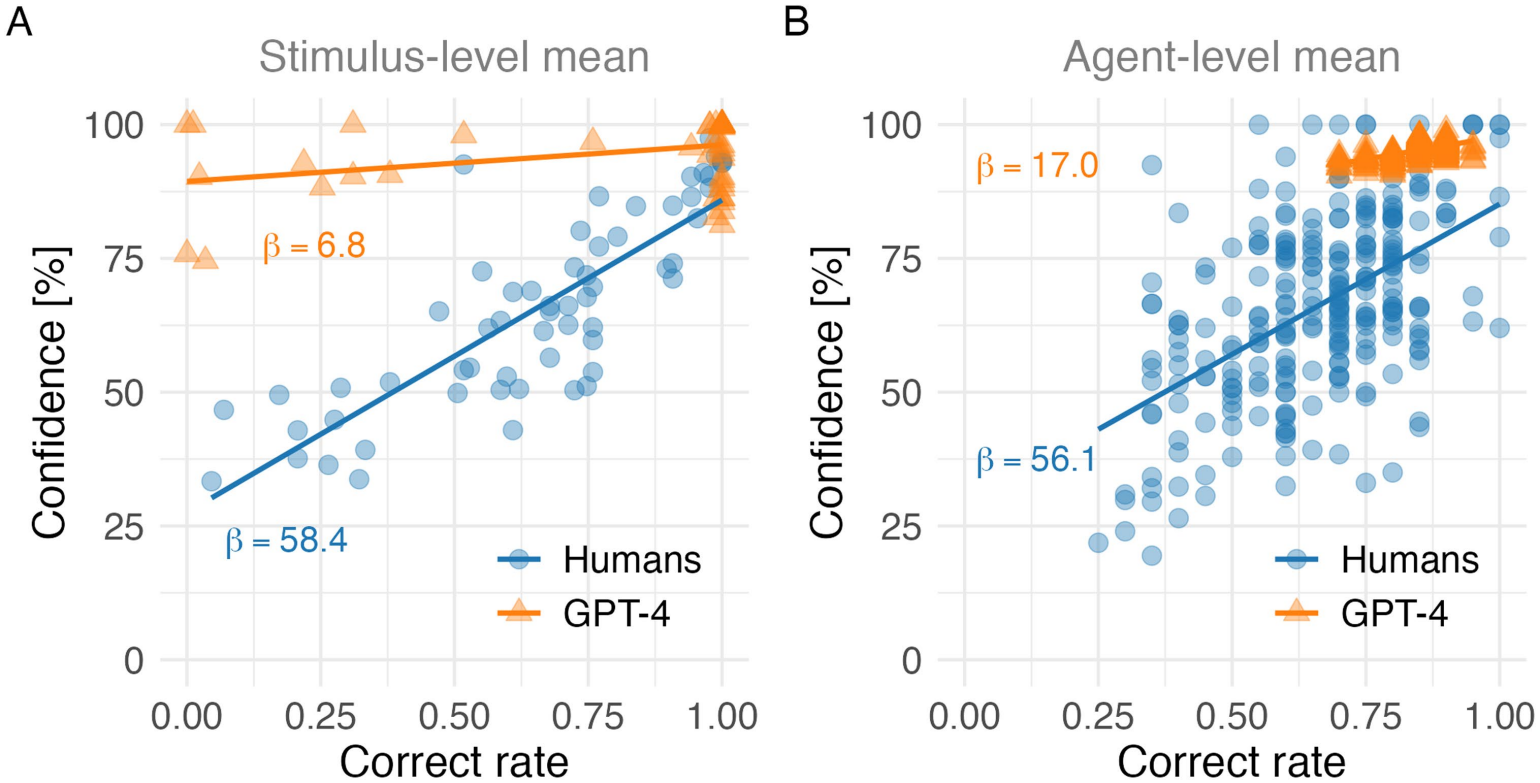
Correlations between correct rate and confidence levels in humans and GPT-4. **(A)** Stimulus-level means. Each point represents the average correct rate and confidence level for a single stimulus (60 points). **(B)** Agent-level means. Each point represents the average correct rate and confidence level for a single agent (*N* = 87). In both panels, solid lines indicate linear regression fits.

Participant-level correct rates and confidence scores for humans and GPT-4 were averaged separately for each of the three conditions (2C, 4C, and OP), and the resulting statistics were compared between the two groups (Result 3.1). Using correct rate (*x*-axis) and confidence (*y*-axis) for humans and GPT-4, we constructed Figure 1 and computed the regression slope and correlation coefficients between these variables (Result 3.2). For humans and GPT-4, responses were classified as correct or incorrect on a task-by-task basis within each of the three conditions, and the corresponding confidence scores were calculated. A mixed-design ANOVA was conducted on mean confidence ratings, with Correctness (correct vs. incorrect) and Cond (2C, 4C, OP) as within-participant factors, and Agent (human vs. GPT-4) as a between-participant factor. Type III sums of squares were used, and participant identity was specified as the random factor (Result 3.3). Metacognitive sensitivity was quantified using Type-2 AUROC, which measures the extent to which confidence ratings discriminate between correct and incorrect responses, independent of confidence bias (Fleming and Lau, 2014). For each participant (*n* = 87) in humans and GPT-4, Type-2 AUROC was computed separately for each task condition (2C, 4C, OP, and All) based on trial-wise accuracy (correct/incorrect) and confidence ratings (0–100) (Result 3.4).

Metacognitive sensitivity was further assessed using meta- J and meta- $J_{2}^{r}$ , information-theoretic measures based on the mutual information between task accuracy and confidence, with meta- $J_{2}^{r}$ indexing metacognitive efficiency by normalizing metacognitive information relative to task performance (correct/incorrect) (Dayan, 2023). Confidence ratings (0–100) were binarized into high versus low confidence using participant-specific thresholds that maximized meta- J , and both measures were computed separately for each task condition (2C, 4C, OP, ALL) for human participants and GPT-4 (Result 3.5). To examine the folded X-pattern, we plotted mean confidence separately for correct and incorrect responses as a function of normalized discriminability. Discriminability was computed as (correct rate—chance level)/(1 − chance level) and divided into four quartiles within each condition (Result 3.6).

All statistical analyses were conducted using R (version 4.5.1; R Core Team, 2025). The analysis scripts are publicly available in the online repository (see Data Availability Statement).

## 3 Results

The twenty general knowledge questions about Japan were posed to human volunteers and GPT-4. One condition required a response using a multiple-choice format with two alternative choices (2C), another used a multiple-choice format with four alternative choices (4C), and the third condition involved an open-ended response (OP). An example of the questions was “What is the largest lake in Japan?” Each question was adjusted in terms of expression depending on the answering condition and options were presented simultaneously in the 2C and 4C conditions. Further details are provided in the Method section and Online Material.

### 3.1 Correct rates and confidence levels

The mean correct rates of the twenty questions were as follows: For humans, they were 0.73 ± 0.11 (mean ± SD), 0.73 ± 0.12, and 0.52 ± 0.14 for 2C, 4C, and OP, respectively: For GPT-4, they were 0.87 ± 0.04, 0.88 ± 0.03, 0.78 ± 0.05, for 2C, 4C, and OP, respectively. For each participant, the correct rate was calculated as the proportion of correct responses across trials within each condition.

These participant-level correct rates were then averaged to obtain the mean correct rates for each condition. A mixed-design ANOVA was conducted on mean correct rates, with Agent (humans vs. GPT-4) as a between-participant factor and Condition (2C, 4C, or OP) as a within-participant factor. Greenhouse–Geisser corrections were applied when the assumption of sphericity was violated.

The results revealed a significant main effect of Agent in which GPT-4 outperformed humans in terms of correct rate [*F*(1, 172) = 236.9, *p* = 3.6 × 10^−34^, $\eta_{G}^{2}$ = 0.496]. The main effect of answering Condition (2C, 4C or OP) also showed significant differences [*F*(1.90, 326.23) = 369.31, *p* = 4.6 × 10^−82^, $\eta_{G}^{2}$ = 0.379]. The interaction effect of the Agent × Condition was statistically significant [*F*(1.90, 326.23) = 78.73, *p* = 5.1 × 10^−21^, $\eta_{G}^{2}$ = 0.086], suggesting that the main effect of condition differed between agents. Given the significant Agent × Condition interaction in correct rates, Bonferroni-corrected *post hoc* comparisons were conducted to further characterize differences between conditions and agents. To examine the interaction further, condition effects were analyzed separately for each agent. In humans, correct rates were significantly lower in the OP condition than in both the 2C (*p* = 7.8 × 10^−53^) and 4C (*p =* 5.7 × 10^−50^) conditions, while no significant difference was found between the 2C and 4C conditions (*p* = 1.00). A similar pattern was observed for GPT-4, with significantly lower correct rates in the OP condition compared to both 2C (*p* = 8.3 × 10^−18^) and 4C (*p* = 8.7 × 10^−17^), and no difference between 2C and 4C (*p* = 1.00). These findings indicate that both humans and GPT-4 exhibit a decline in the correct rate under the OP condition. Agent comparisons within each condition revealed that GPT-4 achieved significantly higher correct rates than humans in all conditions, including 2C (*p* = 4.3 × 10^−22^), 4C (*p* = 4.2 × 10^−22^), and OP (*p* = 2.0 × 10^−36^).

The correct rate analyses revealed robust and systematic differences between humans and GPT-4 across task conditions. Overall, GPT-4 consistently achieved higher correct rates than humans in all conditions, indicating superior task-level performance. This pattern was observed for both multiple-choice formats (2C and 4C) and the open-ended (OP) condition, demonstrating that GPT-4’s advantage in accuracy generalizes across different task structures. Both humans and GPT-4 showed a marked reduction in correct rates in the OP condition relative to the 2C and 4C conditions. This finding indicates that the OP condition was the most challenging for both agents, suggesting a shared difficulty structure across task types. Importantly, the direction of the condition effect was consistent across agents, indicating that humans and GPT-4 are similarly sensitive to relative task difficulties. Despite this shared pattern, the magnitude of the condition effect differed between agents, as reflected by a significant Agent × Condition interaction. In particular, humans exhibited a much steeper decline in performance in the OP condition than GPT-4, suggesting that GPT-4 is more resilient to the demands imposed by open-ended tasks. Together, these results demonstrate that although humans and GPT-4 share a common task-difficulty structure, they differ substantially in overall performance level and in how strongly their performance is modulated by task condition.

The mean confidence levels when participants answered each question were as follows: For humans, they were 72 ± 15% (mean ± SD), 70 ± 15%, and 56 ± 16% for 2C, 4C, and OP conditions, respectively: For GPT-4, they were 95 ± 1%, 97 ± 1%, and 93 ± 1% for 2C, 4C, and OP conditions, respectively. As an analysis of confidence levels, we conducted a mixed-design ANOVA on mean confidence levels, with Agent (humans vs. GPT-4) as a between-participant factor and Condition (2C, 4C, or OP) as a within-participant factor. Greenhouse–Geisser corrections were applied when the assumption of sphericity was violated. The analysis revealed a significant main effect of Agent [*F*(1, 172) = 373.94, *p* = 5.3 × 10^−45^, $\eta_{G}^{2}$ = 0.641], Condition [*F*(1.58, 272.50) = 146.63, *p* = 1.2 × 10^−37^, $\eta_{G}^{2}$ = 0.133], and the interaction between Agent and Condition [*F*(1.58, 272.50) = 78.73, *p* = 1.9 × 10^−23^, $\eta_{G}^{2}$ = 0.076]. Given the significant main effects of Agent and Condition, as well as their interaction on confidence levels, Bonferroni-corrected *post hoc* comparisons were conducted to further characterize these effects. To further examine the significant Agent × Condition interaction, condition effects were analyzed separately for each agent. In humans, confidence was significantly higher in the 2C condition than in the 4C condition (*p* = 0.035), higher in 2C than in OP (*p* = 5.3 × 10^−42^), and higher in 4C than in OP (*p* = 7.4 × 10^−31^). In GPT-4, confidence did not differ significantly between the 2C and 4C conditions (*p* = 0.070), nor between the 2C and OP conditions (*p* = 0.12). However, confidence was significantly higher in the 4C condition than in the OP condition (*p* = 0.0051). Finally, agent comparisons within each condition revealed that GPT-4 reported significantly higher confidence than humans in all conditions, including 2C (*p* = 1.6 × 10^−32^), 4C (*p* = 1.8 × 10^−35^), and OP (*p* = 8.8 × 10^−51^).

The confidence level analyses revealed robust and systematic differences between humans and GPT-4 across all task conditions. Overall, GPT-4 consistently reported substantially higher confidence than human participants, regardless of task format, indicating a strong agent-level difference in the absolute level of confidence. This pattern was observed in all conditions, including 2C, 4C, and OP, demonstrating that GPT-4’s elevated confidence generalizes across different task structures. In humans, confidence levels were strongly modulated by task condition, decreasing monotonically as task difficulty increased. Specifically, confidence was highest in the 2C condition, intermediate in the 4C condition, and lowest in the OP condition. This graded pattern suggests that human confidence systematically reflects perceived task difficulty and internal uncertainty. In contrast, GPT-4’s confidence showed minimal modulation by task condition. Although a specific difference was observed between the 4C and OP conditions, confidence remained uniformly high across all conditions, indicating a restricted dynamic range. This lack of graded adjustment suggests that GPT-4 does not down-regulate confidence in response to increasing task difficulty in the same way as humans. Importantly, the significant Agent × Condition interaction demonstrates that humans and GPT-4 differ not only in the overall level of confidence but also in how confidence is shaped by task structure. Whereas human confidence flexibly adapts to changes in task difficulty, GPT-4’s confidence remains largely invariant across conditions.

### 3.2 Confidence–accuracy relationships at the agent and stimulus levels

Confidence level would reflect the difficulty of questions and the likelihood of correct answers, thus helping build a robust basis for cognition and behavior. Confidence levels reflecting changes in task difficulty (correct rates) have been regarded as evidence of metacognition. In view of this role of confidence, we analyzed the relationship between correct rates and confidence levels at two distinct levels: the agent level (individual differences) and the stimulus level (item difficulty). Specifically, we calculated the means separately for each level: (1) For the stimulus-level analysis, correct rates and confidence levels were averaged across participants for each question. (2) For the agent-level analysis, they were averaged across questions for each agent (i.e., each participant or session). We then examined the regression slopes and Pearson correlations between correct rates and confidence levels for both measures.

Figure 1 shows the relationship between correct rates and confidence levels for 60 questions in the 2C, 4C, and OP conditions in humans and GPT-4, based on the stimulus-level mean (Figure 1A) and the agent-level mean (Figure 1B). Regression analyses revealed that the relationship between correct rates and confidence levels was significantly stronger for humans than for GPT-4, as indicated by a steeper regression slope (*Δβ* =β{GPT-4}−*β* {humans} at both the stimulus-level mean (*Δβ* = −51.55, *p* = 6.2 × 10^−16^, *t*(116) = −9.40) and the agent-level mean (*Δβ* = −39.12, *p* = 9.3 × 10^−4^, *t*(518) = −3.33). Across both the stimulus-level and agent-level means, humans show larger regression slopes (*β*) than GPT-4, suggesting a broader dynamic range of the correct rate and the confidence level in humans.

At the stimulus-level mean, Pearson correlation coefficients between correct rates and confidence levels were 0.83 (*p* = 2.9 × 10^−16^) for humans and 0.33 (*p* = 0.0098) for GPT-4. For the agent-level mean, the corresponding coefficients were 0.53 (*p* = 2.1 × 10^−20^) for humans and 0.57 (*p* = 1.3 × 10^−24^) for GPT-4. To compare whether the correlation coefficients differed between humans and the LLM, we applied Fisher’s z transformation as follows. The test for the difference between correlation at the stimulus-level mean yielded *z* = 4.51 (*p* = 6.4 × 10^−6^) (*p* < 0.0001), whereas the corresponding test at the agent-level mean yielded *z* = −0.37 (*p* = 0.71). Notably, the confidence level of GPT-4 for a question with a correct rate of 0 was above 75% at the stimulus-level mean and above 88% at the agent-level mean.

At the agent level, no difference was observed between humans and GPT-4 in the strength of the correlation between the correct rate and the confidence level. However, the regression slope differed significantly, with humans showing a steeper slope. This suggests that although the level of correlation (i.e., linearity) is comparable, humans adjust their confidence more sensitively to changes in accuracy, effectively using a wider dynamic range than GPT-4.

### 3.3 Comparison of confidence levels in correct and incorrect answers

If confidence levels reflected the ability to answer correctly, it was expected that the confidence levels would be higher when the participants responded correctly. We compared the confidence levels separately for correct answers and incorrect answers within each agent.

**Figure 2:**
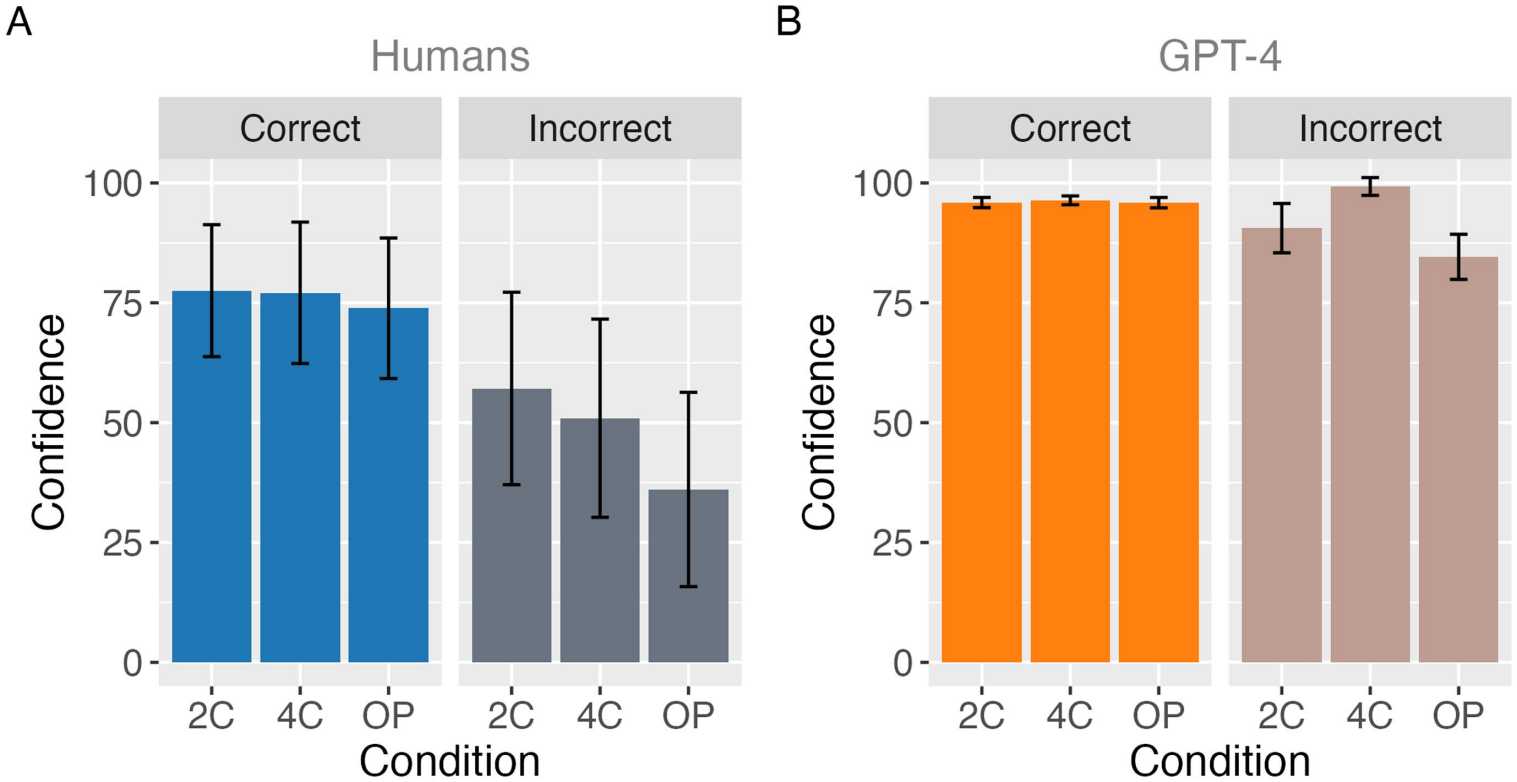
Confidence in correct and incorrect answers in humans **(A)** and in GPT-4 **(B)**. Bars indicate mean confidence ratings, and error bars represent ±1 SD across agents.

In humans, confidence levels for correct answers were 78 ± 14% (mean ± SD), 77 ± 15%, and 74 ± 15% for the 2C, 4C, and OP conditions, respectively. For incorrect answers, confidence levels were 57 ± 20%, 51 ± 20%, and 36 ± 20%, respectively (Figure 2A). In GPT-4, confidence levels for correct answers were 96 ± 1%, 96 ± 1%, and 96 ± 1% among the 2C, 4C, and OP conditions, respectively, while for incorrect answers, they were 91 ± 5%, 99 ± 1%, and 85 ± 5% (Figure 2B). A mixed-design ANOVA was conducted on mean confidence levels, with Agent (humans vs. GPT-4) as a between-participant factor, and Correctness (correct vs. incorrect) and Condition (2C, 4C, or OP) as within-participant factors. Greenhouse–Geisser corrections were applied when the assumption of sphericity was violated. There was a significant main effect of Agent [*F*(1, 169) = 512.85, *p* = 4.6 × 10^−53^, $\eta_{G}^{2}$ = 0.628], Correctness [*F*(1, 169) = 510.79, *p* = 5.9 × 10^−53^, $\eta_{G}^{2}$ = 0.303], and Condition [*F*(1.7, 289.20) = 86.18, *p* = 6.8 × 10^−27^, $\eta_{G}^{2}$ = 0.089]. All two-way interactions between Agent and Correctness [*F*(1, 169) = 263.30, *p* = 2.7 × 10^−36^, $\eta_{G}^{2}$ = 0.183], Agent and Condition [*F*(1.7, 289.20) = 25.77, *p* = 7.2 × 10^−10^, $\eta_{G}^{2}$ = 0.028], Correctness and Condition [*F*(1.8, 303.87) = 95.00, *p* = 2.3 × 10^−30^, $\eta_{G}^{2}$ = 0.057], and the three-way interaction between Agent, Correctness, and Condition [*F*(1.8, 303.87) = 25.68, *p* = 3.2 × 10^−10^, $\eta_{G}^{2}$ = 0.016] were statistically significant. Bonferroni-corrected *post hoc* comparisons were conducted to further characterize the significant effects observed in confidence ratings. Across all Conditions, GPT-4 reported significantly higher confidence than humans for both Correct and Incorrect responses. For Correct responses, confidence was higher for GPT-4 than for humans in the 2C (*p* = 6.2 × 10^−27^), 4C (*p* = 3.7 × 10^−26^), and OP (*p* = 8.6 × 10^−31^) conditions. The same pattern was observed for Incorrect responses, with GPT-4 showing higher confidence than humans in 2C (*p* = 1.1 × 10^−32^), 4C (*p* = 8.8 × 10^−51^), and OP (*p* = 2.2 × 10^−54^). Within the Human group, confidence varied significantly across Conditions. For Correct responses, confidence was significantly lower in the OP condition than in both 2C (*p* = 0.0004) and 4C (*p* = 0.0079), whereas no significant difference was observed between 2C and 4C (*p* = 1.0). For Incorrect responses, confidence also differed across Conditions, with confidence highest in 2C, followed by 4C, and lowest in OP (2C *vs.* 4C: *p* = 9.2 × 10^−6^; OP *vs.* 2C: *p* = 1.3 × 10^−24^; OP vs. 4C: *p* = 2.2 × 10^−16^). In contrast, for GPT-4, confidence for Correct responses did not differ significantly across Conditions (all *p*s = 1.0). However, for Incorrect responses, confidence varied reliably by Condition, being highest in 4C, followed by 2C, and lowest in OP (2C *vs.* 4C: *p* = 5.6 × 10^−10^; OP *vs.* 2C: *p* = 2.5 × 10^−3^; OP *vs.* 4C: *p* = 3.0 × 10^−15^). Finally, confidence was significantly higher for Correct than for Incorrect responses in humans across all Conditions (2C: *p* = 3.4 × 10^−34^; 4C: *p* = 2.7 × 10^−45^; OP: *p* = 7.1 × 10^−61^). A similar pattern was observed for GPT-4 in the 2C (*p* = 3.5 × 10^−5^) and OP (*p* = 6.4 × 10^−13^) conditions. In contrast, in the 4C condition, GPT-4 exhibited significantly higher confidence for Incorrect than for Correct responses (*p* = 0.026).

These results demonstrate systematic and qualitative differences in confidence behavior between humans and GPT-4. Across all task conditions and irrespective of correctness, GPT-4 consistently reported higher confidence than human participants, indicating a robust agent-related difference in overall confidence level. In contrast, human confidence reliably reflected response correctness, with confidence being significantly higher for correct than for incorrect responses across all conditions. Human confidence further exhibited clear condition dependence, decreasing as task difficulty increased, with the lowest confidence observed in the OP condition for both correct and incorrect responses. This pattern suggests that human confidence is flexibly modulated by task structure in addition to response accuracy. By comparison, GPT-4’s confidence for correct responses remained uniformly high and did not vary across conditions, indicating a lack of adjustment to task difficulty when responses were correct. Importantly, GPT-4 showed a distinct and condition-specific pattern for incorrect responses: confidence was highest in the 4C condition, followed by the 2C condition, and lowest in the OP condition. This ordering contrasts with that observed in humans and reveals a qualitative divergence in how confidence relates to task structure during incorrect answers.

### 3.4 Type-2 AUROC

Type-2 AUROC for All, 2C, 4C and OP conditions in humans and GPT-4 were calculated (Table 1), where each condition was obtained by the Agent-level mean. The Type-2 AUROC was evaluated by the conditional probability of confidence conditioned on correct, and the conditional probability of confidence conditioned on incorrect. In humans, Type-2 AUROC was 0.76 ± 0.14 (mean ± SD) for the All condition, 0.70 ± 0.14 for the 2C condition, 0.76 ± 0.13 for the 4C condition, and 0.81 ± 0.12 for the OP condition, respectively. On the other hand, Type-2 AUROC in GPT-4 was 0.63 ± 0.20 (mean ± SD) for the All condition, 0.73 ± 0.15 for the 2C condition, 0.40 ± 0.07 for the 4C condition, and 0.78 ± 0.09 for the OP condition, respectively.

**Table 1:** Type-2 AUROC for each All, 2C, 4C, and OP condition in humans and GPT-4.

Agent	Condition	Type-2 AUROC ± SD
Humans	All (2C, 4C, OP)	0.76 ± 0.14
	2C	0.70 ± 0.14
	4C	0.76 ± 0.13
	OP	0.81 ± 0.12
GPT-4	All (2C, 4C, OP)	0.63 ± 0.20
	2C	0.73 ± 0.15
	4C	0.40 ± 0.07
	OP	0.78 ± 0.09

As an analysis of Type-2 AUROC, we conducted a mixed-design ANOVA on the agent-level mean AUROC, with Agent (humans vs. GPT-4) as a between-participant factor and Condition (2C, 4C, or OP) as a within-participant factor. Greenhouse–Geisser corrections were applied when the assumption of sphericity was violated. The analysis revealed a significant main effect of Agent [*F*(1, 165) = 93.73, *p* = 7.8 × 10^−18^, $\eta_{G}^{2}$ = 0.210], Condition [*F*(1.84, 303.76) = 179.85, *p* = 1.6 × 10^−49^, $\eta_{G}^{2}$ = 0.367], and the interaction between Agent and Condition [*F*(1.84, 303.76) = 161.07, *p* = 7.9 × 10^−46^, $\eta_{G}^{2}$ = 0.34]. Given the significant interaction, Bonferroni-corrected *post hoc* pairwise comparisons were performed. When averaged across Agents, comparisons among Conditions showed that Type-2 AUROC was significantly higher in 2C than in 4C (*p* = 1.9 × 10^−22^), higher in OP than in 2C (*p* = 1.3 × 10^−8^), and higher in OP than in 4C (*p* = 1.0 × 10^−50^). For the main effect of Agent averaged across Conditions, humans showed significantly higher Type-2 AUROC than GPT-4 (*p* = 7.8 × 10^−18^). To further examine the interaction, Condition effects were analyzed separately for each Agent. For humans, Type-2 AUROC was significantly higher in OP than in 2C (*p* = 6.4 × 10^−8^) and 4C (*p* = 6.7 × 10^−4^). 4C showed higher AUROC than 2C (*p* = 0.0052). For GPT-4, Type-2 AUROC was significantly higher in OP than in 2C (*p* = 0.016) and 4C (*p* = 4.2 × 10^−64^). 2C showed higher Type-2 AUROC than 4C (*p* = 2.0 × 10^−45^). Finally, Agent comparisons within each Condition revealed that humans showed significantly higher Type-2 AUROC than GPT-4 in the 4C condition (*p* = 2.7 × 10^−52^), whereas no significant differences between Agents were observed in the 2C (*p* = 0.22) or OP (*p* = 0.059) conditions.

The Type-2 AUROC analyses revealed pronounced and systematic differences in metacognitive sensitivity between humans and GPT-4. Overall, humans exhibited significantly higher Type-2 AUROC than GPT-4 when averaged across task conditions, indicating a greater ability to discriminate correct from incorrect responses on the basis of confidence. In addition, Type-2 AUROC was strongly modulated by task condition, demonstrating that metacognitive sensitivity is not invariant across task structures. Importantly, the effect of condition differed qualitatively between agents, as reflected in a robust Agent × Condition interaction. In humans, Type-2 AUROC increased monotonically across conditions, with the highest sensitivity observed in the open-ended (OP) condition, followed by the four-choice (4C) and two-choice (2C) conditions. This pattern indicates that human metacognitive sensitivity is maintained—and even enhanced—under more or less difficult task contexts. In contrast, GPT-4 showed a non-monotonic and condition-specific pattern of Type-2 AUROC. Although GPT-4 exhibited relatively high sensitivity in the OP and 2C conditions, its Type-2 AUROC dropped markedly in the 4C condition. As a result, a clear agent difference emerged specifically in the 4C condition, where humans showed substantially higher Type-2 AUROC than GPT-4, whereas no significant differences between agents were observed in the 2C or OP conditions.

### 3.5 Meta- J and meta- $J_{2}^{r}$

Meta- J and meta- $J_{2}^{r}$ were evaluated and described for the All, 2C, 4C and OP conditions in humans and GPT-4 (Table 2), where each condition was obtained by the Agent-level mean. In humans, meta- J between task accuracy (correct/incorrect) and confidence levels were 0.19 ± 0.11, 0.16 ± 0.13, 0.21 ± 0.14, and 0.34 ± 0.20 for the All, 2C, 4C and OP conditions, respectively. On the other hand, the meta- J in GPT-4 was 0.06 ± 0.03, 0.14 ± 0.09, 0.048 ± 0.023, and 0.21 ± 0.13 for the All, 2C, 4C, and OP conditions, respectively.

**Table 2:** Meta- J and meta- $J_{2}^{r}$ were calculated and described for each All, 2C, 4C, and OP condition in humans and GPT-4.

Agent	Condition	Meta*-* J ± SD	Meta- $J_{2}^{r}\;$ ± SD
Humans	All (2C, 4C, OP)	0.19 ± 0.11	0.21 ± 0.12
	2C	0.16 ± 0.13	0.21 ± 0.18
	4C	0.21 ± 0.14	0.27 ± 0.18
	OP	0.34 ± 0.20	0.37 ± 0.21
GPT-4	All (2C, 4C, OP)	0.061 ± 0.031	0.097 ± 0.048
	2C	0.14 ± 0.09	0.27 ± 0.17
	4C	0.048 ± 0.023	0.090 ± 0.037
	OP	0.21 ± 0.13	0.29 ± 0.17

As an analysis of meta- J , we conducted a mixed-design ANOVA on the agent-level mean meta- J , with Agent (humans vs. GPT-4) as a between-participant factor and Condition (2C, 4C, or OP) as a within-participant factor. Greenhouse–Geisser corrections were applied when the assumption of sphericity was violated. The analysis revealed a significant main effect of Agent [*F*(1, 172) = 55.68, *p* = 4.0 × 10^−12^, $\eta_{G}^{2}$ = 0.135], Condition [*F*(1.76, 301.89) = 85.09, *p* = 2.5 × 10^−27^, $\eta_{G}^{2}$ = 0.204], and the interaction between Agent and Condition [*F*(1.76, 301.89) = 18.11, *p* = 1.8 × 10^−7^, $\eta_{G}^{2}$ = 0.052]. Bonferroni-corrected *post hoc* comparisons were conducted to further characterize the significant effects observed in meta- J . When averaged across Agents, meta- J differed reliably across Conditions: meta- J was higher in the OP condition than in both 2C (*p* = 1.0 × 10^−15^) and 4C (*p* = 3.8 × 10^−23^), whereas the difference between 2C and 4C did not reach significance (*p* = 0.052). When averaged across Conditions, humans exhibited significantly higher meta- J than GPT-4 (*p* = 4.0 × 10^−12^). However, follow-up comparisons revealed that this Agent difference was Condition-dependent. Specifically, humans showed higher meta- J than GPT-4 in the 4C (*p* = 1.3 × 10^−20^) and OP (*p* = 1.1 × 10^−6^) conditions, whereas no significant difference between Agents was observed in the 2C condition (*p* = 0.27). Condition effects further differed between Agents. For humans, meta- J varied significantly across all Conditions, with higher meta- J in OP than in 2C (*p* = 4.2 × 10^−16^) and 4C (*p* = 1.7 × 10^−11^), and higher meta- J in 4C than in 2C (*p* = 0.003). For GPT-4, meta- J also differed across Conditions but followed a distinct pattern: meta- J was higher in 2C than in 4C (*p* = 6.8 × 10^−10^), higher in OP than in 2C (*p* = 0.0013), and higher in OP than in 4C (*p* = 5.2 × 10^−16^).

The meta- J analyses revealed systematic differences in metacognitive sensitivity between humans and GPT-4. Overall, humans exhibited significantly higher meta- J than GPT-4, indicating greater sensitivity of confidence to response correctness at the aggregate level (All). However, this agent difference was not uniform across task conditions: no significant difference was observed in the simple two-choice (2C) condition, whereas humans showed reliably higher meta- J than GPT-4 in the more difficult four-choice (4C) and open-ended (OP) conditions. Meta- J was strongly modulated by task condition in both agents, but the pattern of modulation differed qualitatively. In humans, meta- J varied systematically across all conditions, with the highest values observed in the OP condition, followed by 4C and then 2C, indicating that human metacognitive sensitivity was maintained—and even enhanced—under more difficult tasks. In contrast, GPT-4 showed a non-monotonic and condition-specific pattern of meta- J , with markedly reduced sensitivity in the 4C condition and higher values in the 2C and OP conditions.

The meta- $J_{2}^{r}$ between task accuracy (correct/incorrect) and confidence level were 0.21 ± 0.12, 0.21 ± 0.18, 0.27 ± 0.18, and 0.37 ± 0.21 for the All, 2C, 4C and OP conditions, respectively. In GPT-4, meta- $J_{2}^{r}$ were 0.097 ± 0.048, 0.27 ± 0.17, 0.090 ± 0.037, and 0.29 ± 0.17 for the All, 2C,4C and OP conditions, respectively.

As an analysis of meta- $J_{2}^{r}$ , we conducted a mixed-design ANOVA on the agent-level mean meta- $J_{2}^{r}$ , with Agent (humans *vs.* GPT-4) as a between-participant factor and Condition (2C, 4C, or OP) as a within-participant factor. Greenhouse–Geisser corrections were applied when the assumption of sphericity was violated. The analysis revealed a significant main effect of Agent [*F*(1, 169) = 14.43, *p* = 2.0 × 10^−4^, $\eta_{G}^{2}$ = 0.036], Condition [*F*(1.86, 313.52) = 42.20, *p* = 5.0 × 10^−16^, $\eta_{G}^{2}$ = 0.123], and the interaction between Agent and Condition [*F*(1.86, 313.52) = 31.08, *p* = 2.4 × 10^−12^, $\eta_{G}^{2}$ = 0.094]. Bonferroni-corrected *post hoc* comparisons were conducted to further examine differences in meta- $J_{2}^{r}$ . When averaged across Agents, meta- $J_{2}^{r}$ differed significantly across Conditions. Meta- $J_{2}^{r}$ was higher in 2C than in 4C (*p* = 4.6 × 10^−4^), higher in 2C than in OP (*p* = 4.1 × 10^−6^), and higher in 4C than in OP (*p* = 1.1 × 10^−16^). When averaged across Conditions, humans exhibited significantly higher meta- $J_{2}^{r}$ than GPT-4 (*p* = 2.0 × 10^−4^). However, this Agent difference varied by Condition. In the 2C condition, GPT-4 showed higher meta- $J_{2}^{r}$ than human (*p* = 0.0054). In contrast, humans showed higher meta- $J_{2}^{r}$ than GPT-4 in the 4C (*p* = 1.6 × 10^−17^) and OP (*p* = 0.0087) conditions. Condition effects also differed between Agents. For humans, meta- $J_{2}^{r}$ was higher in 4C than in 2C (*p* = 0.0022), higher in OP than in 2C (*p* = 4.9 × 10^−9^), and higher in OP than in 4C (*p* = 0.0001). For GPT-4, meta- $J_{2}^{r}$ was higher in 2C than in 4C (*p* = 1.1 × 10^−15^) and higher in OP than in 4C (*p* = 7.3 × 10^−16^), whereas no significant difference was observed between 2C and OP (*p* = 1).

The meta- $J_{2}^{r}$ analyses revealed systematic differences in metacognitive efficiency between humans and GPT-4. Overall, humans exhibited significantly higher meta- $J_{2}^{r}\;$ than GPT-4 when averaged across task conditions, indicating greater efficiency in translating task accuracy information into confidence judgements. However, this agent-level difference was not uniform across conditions and was strongly modulated by task structure. Task condition exerted a robust effect on meta- $J_{2}^{r}$ in both agents, but with qualitatively different patterns. In humans, meta- $J_{2}^{r}$ varied reliably across all conditions, with the highest efficiency observed in the OP condition, followed by the 4C condition, and the lowest efficiency in the 2C condition. This pattern indicates that human metacognitive efficiency is flexibly adjusted as task difficulty changes and that human metacognitive sensitivity was maintained—and even enhanced—under more difficult tasks. In contrast, GPT-4 exhibited a distinct and less stable pattern of metacognitive efficiency. GPT-4 showed higher meta- $J_{2}^{r}$ than humans in the simple 2C condition, indicating highly efficient use of available information in this context. However, in the more difficult 4C and OP conditions, humans exhibited significantly higher meta- $J_{2}^{r}$ than GPT-4. Moreover, GPT-4’s meta- $J_{2}^{r}$ did not differ between the 2C and OP conditions, suggesting a lack of graded adjustment of efficiency across task structures.

### 3.6 Folded X-pattern

**Figure 3:**
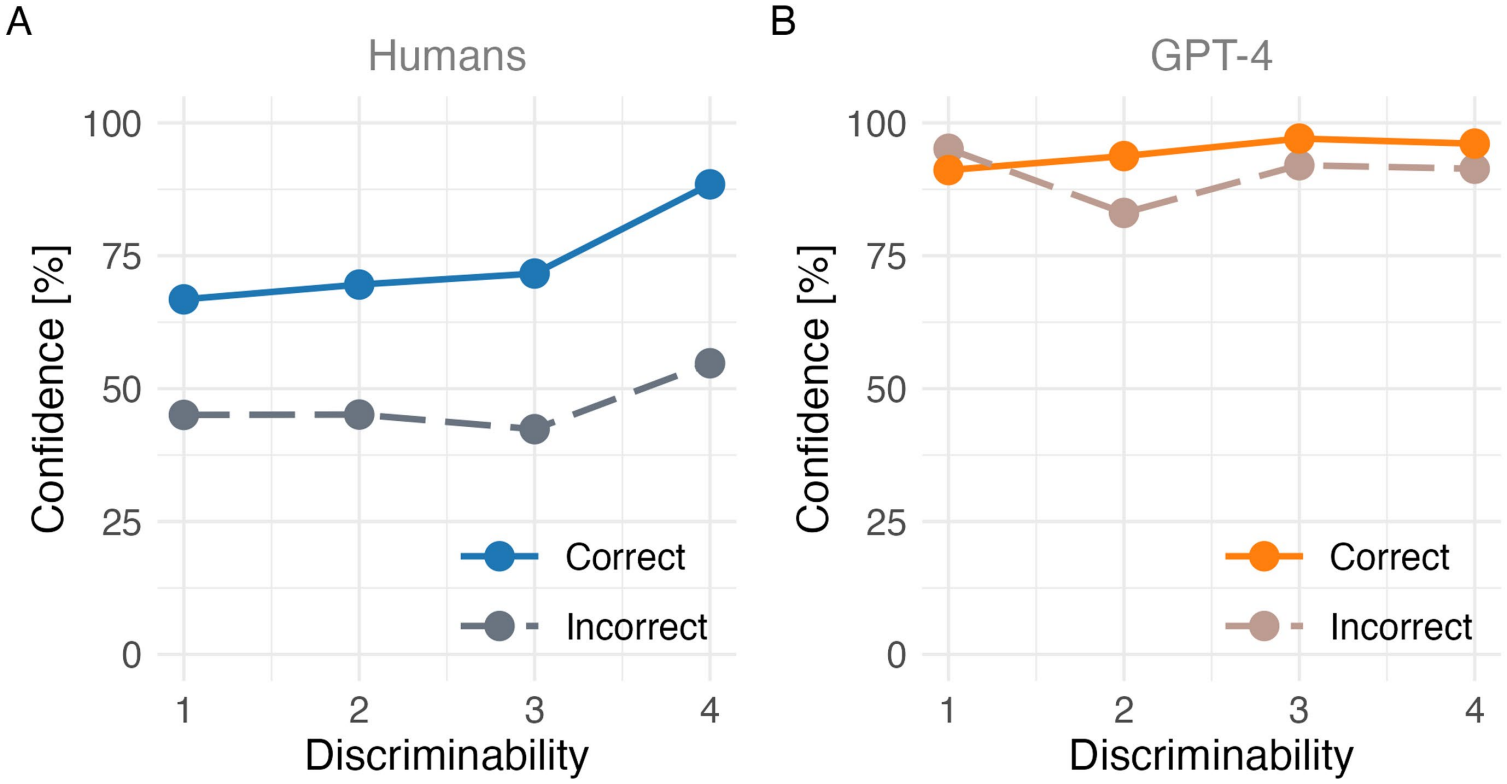
Folded X-pattern made of correct/incorrect answers in humans **(A)** and in GPT-4 **(B)**.

Line plots of correct rates (discriminability, *x*-axis) and confidence levels (*y*-axis) for each of the correct and incorrect answers were drawn for 60 questions in 2C, 4C and OP conditions in humans (Figure 3A) and GPT-4 (Figure 3B), illustrating what is known as the folded X-pattern. Normalized discriminability was defined as (correct rate − chance level) / (1 − chance level), which we then divided into four quartiles. Note that the chance level probabilities were 0.5, 0.25, and 0 for 2C, 4C, and OP conditions, respectively.

In humans, confidence for correct responses generally increased with discriminability, whereas confidence for incorrect responses tended to be lower, resulting in a separation between correct and incorrect curves. Notably, this separation was not strictly monotonic, as confidence for incorrect responses increased in the highest discriminability quartile. In contrast, GPT-4 showed condition-dependent distortions of this separation, with reduced or altered differentiation between correct and incorrect responses across task conditions. These findings revealed a partial separation between confidence for correct and incorrect responses in humans, whereas this separation was weaker and condition-dependent in GPT-4. Notably, deviations from the ideal folded X-pattern were observed, particularly in high-discriminability incorrect responses, highlighting differences in how confidence relates to performance across agents.

## 4 Discussion

Result 3.1 about Correct Rate reveals that GPT-4 consistently outperformed humans across all task conditions, indicating superior task-level accuracy. Importantly, both humans and GPT-4 exhibited similar patterns of task difficulty, with the lowest performance observed in the open-ended (OP) condition. This shared difficulty structure suggests that the relative cognitive demands imposed by the different task formats were broadly comparable across agents. However, the magnitude of the performance decline in the OP condition differed between agents, with humans showing a steeper drop than GPT-4. This dissociation suggests that although humans and GPT-4 respond to task difficulty in similar directions, their robustness to increasing task demands differs substantially. Result 3.1 about Confidence Level indicate that confidence plays qualitatively different functional roles in humans and GPT-4. In humans, confidence appears to operate as a graded signal of uncertainty that tracks task difficulty, whereas in GPT-4, confidence is persistently high and weakly sensitive to task demands, suggesting a fundamentally different mechanism of confidence generation across biological and artificial agents. Result 3.2 suggests that human confidence functions as a graded internal signal that quantitatively tracks task difficulty and performance, whereas GPT-4’s confidence reflects a more rigid or saturated mapping that lacks fine-grained adjustment. This qualitative difference helps explain why GPT-4 can show moderate correlations between confidence and accuracy while simultaneously exhibiting low metacognitive sensitivity and efficiency in other analyses. More broadly, the Figure 1 results highlight that apparent alignment between confidence and accuracy in LLMs may mask important differences in how confidence information is generated and utilized. Result 3.3 indicates that while human confidence systematically integrates information about both correctness and task condition, GPT-4’s confidence exhibits reduced sensitivity to correctness and condition-dependent reversals during incorrect responses, highlighting fundamental differences in confidence generation across agents. Result 3.4 indicates that GPT-4 can achieve human-comparable metacognitive sensitivity in certain task contexts, but this sensitivity does not generalize robustly across task structures.

By contrast, human metacognitive sensitivity, as indexed by Type-2 AUROC, appears more stable and consistently aligned with task demands, highlighting a qualitative difference in how confidence information is used to monitor performance across biological and artificial agents. Result 3.5 about meta- J indicates that while GPT-4 can exhibit human-comparable metacognitive sensitivity in simple decision contexts, its meta- J is highly dependent on task structure. Result 3.5 about meta- $J_{2}^{r}\;$ demonstrates a significant Agent × Condition interaction in metacognitive efficiency. While GPT-4 can exhibit high efficiency in simple decision contexts, its efficiency is strongly condition-specific and does not generalize robustly across task structures. By comparison, human metacognitive sensitivity and efficiency appear more robust across task contexts, highlighting a qualitative difference in how confidence information is linked to performance across biological and artificial agents. Result 3.6 reveals that human confidence partially tracks internal discriminability, exhibiting a characteristic separation between correct and incorrect responses despite deviations at high discriminability levels. In contrast, GPT-4 showed weaker and condition-dependent separations, suggesting that its confidence does not consistently encode internal evidence strength but instead reflects structural properties of its output distributions.

Taken together, these converging results reveal a fundamental dissociation between performance and metacognition in GPT-4. Although GPT-4 consistently achieves high levels of task accuracy, its confidence does not function as a graded internal signal of uncertainty in the same way as human confidence. In humans, confidence appears to encode an internal estimate of success probability that flexibly integrates information about task difficulty, discriminability, and correctness. In contrast, GPT-4’s confidence remains uniformly high, weakly sensitive to accuracy, and strongly shaped by task structure, suggesting that it reflects surface-level properties of its output distribution rather than a robust internal monitoring process.

These findings carry important implications for the interpretation of confidence in LLMs. High confidence in an LLM should not be equated with human-like metacognitive capability. Apparent metacognitive signatures—such as positive correlations between confidence and accuracy or high Type-2 AUROC in specific conditions—may arise as artifacts of output geometry rather than from genuine internal uncertainty monitoring. This distinction is critical for applications in which LLMs are expected to assess their own reliability, such as decision support systems (Handler et al., 2024), human–AI collaboration (Vaccaro et al., 2024), and safety-critical domains (Yampolskiy, 2018).

In sum, our results demonstrate that humans and GPT-4 differ not only in the level of confidence they report, but also in the functional role that confidence plays. Human confidence serves as a flexible and informative signal of internal uncertainty, whereas GPT-4’s confidence appears compressed, condition-dependent, and weakly coupled to performance. These qualitative differences underscore the importance of distinguishing between task-level capability and metacognitive capability and caution against interpreting high confidence in artificial agents as evidence of human-like self-monitoring abilities.

An important consideration in metacognitive research is the extent to which findings generalize across different levels of task difficulty, given the well-known task dependence of metacognitive processes. Although our tasks consisted of trivia-style questions, they exhibited substantial natural variation in difficulty. As shown in Figure 1A, correct rates spanned a wide range, from near floor (0%) to near ceiling (100%), indicating that the task set was not restricted to a narrow difficulty range. Furthermore, we explicitly addressed difficulty variation by introducing a standardized measure of task difficulty—discriminability—which accounts for the number of response alternatives (Figure 3). This measure provides a principled way to normalize task difficulty across items and allows for meaningful comparisons across agents. Using this standardized difficulty metric, we observed the folded X-pattern in human participants, whereas this characteristic pattern was absent in GPT-4. Together, these results suggest that our conclusions are not driven by a restricted or homogeneous range of task difficulty. Rather, they reflect systematic differences in how humans and GPT-4 express confidence across varying levels of difficulty. Therefore, we believe that the observed dissociation between human and GPT-4 metacognitive patterns cannot be attributed to insufficient difficulty variation in the task set.

Cash et al. (2025) demonstrated, using various LLMs and tasks, that LLMs exhibited higher metacognitive accuracy than humans. In contrast, Griot et al. (2025) showed that multiple LLMs lacked metacognitive ability when responding to medical questions. However, what these two studies had in common was that LLMs tended to be overconfident. In our study, we introduced a novel approach by manipulating uncertainty through general questions with open-ended, four-choice, and two-choice formats. As a result, we found that LLMs were overconfident and appeared to have context-dependent, unstable and lacking robust metacognitive ability compared to humans.

Our results were consistent with those of Griot et al. (2025). They addressed medical questions, did not compare LLMs with humans, and also presented LLMs with questions in which the correct answer was intentionally omitted from the choices, providing “I do not know or cannot answer” or “None of the above” options. In comparison, our study compares the metacognitive capabilities of LLMs with humans in two choices, and four choices, and open-ended questions.

In general, metacognitive sensitivity is known to be affected by metacognitive bias (e.g., shy people are always less confident, Fleming and Lau, 2014). Our study differs from Griot et al. (2025) in that we calculated meta- J , meta- $J_{2}^{r}$ and Type-2 AUROC to exclude metacognitive bias and compared metacognitive sensitivity between humans and LLMs.

Taken together, our findings indicate that, relative to humans, LLMs show overconfidence, pronounced context dependence, and instability in their confidence judgements, suggesting a lack of robust metacognitive ability, or at least that its metacognitive processes differ substantially from those of humans.

Our study has several limitations. The questions in this study were limited to the context of a single culture—Japan. Future research should examine LLMs by asking questions across a wider range of cultural contexts and in multiple languages.

Using prompt-based evaluations to probe LLMs’ metacognition, we have argued that LLMs may lack metacognitive ability; however, we must be cautious about whether the observed patterns in this GPT-4 experiment actually reflect metacognition or are merely calibration artifacts. More broadly, whether concepts developed for humans apply to LLMs is under active examination across a range of constructs, including theory of mind (Strachan et al., 2024) and the Big Five personality traits (Serapio-García et al., 2025). Responses of GPT-4 are simply texts generated by next token predictions induced by prompts, and do not necessarily reflect genuine metacognitive functions or lack thereof. In order to build an explainable AI (XAI) (Dwivedi et al., 2023), however, giving an accurate answer on confidence level is necessary, no matter what kind of models one may assume for metacognition.

Another limitation of our study is that we conducted the experiment only with GPT-4 among the available LLMs. Therefore, our results should be interpreted with caution and cannot necessarily be generalized to other models. Nevertheless, previous studies employing a range of LLMs under diverse experimental conditions have also reported that LLMs tend to be overconfident (Cash et al., 2025; Griot et al., 2025), suggesting that our finding with GPT-4 is consistent with this broader trend.

The tendency for LLMs to exhibit overconfidence may also result from RLHF-based post-training (Reinforcement Learning from Human Feedback, Ouyang et al., 2022). For example, OpenAI et al. (2024a) and Kalai et al. (2025) argue that plausible responses to multiple-choice queries were worse calibrated after RLHF-based post-training than before.

Regarding alignment and safety, hallucination has become a major issue, as LLMs sometimes persist in giving false answers or fabricate information in response to human instructions. To address this, systems have been developed that enable LLMs to cite sources and explain their reasoning. In our study, we examined whether GPT-4, which was a state-of-the-art LLM at the time of the experiment, lowers its confidence when it is wrong, as a way to assess its metacognitive ability. The results suggested that, at present, robust metacognition may be lacking in this model.

Whether metacognition can be learned over time remains uncertain. Even in human adolescents, it has been shown that learning metacognition through prompts is difficult (Klar et al., 2024). Thus, whether future models will truly be capable of metacognition is still unknown. Moreover, whether LLMs possessing metacognition would actually contribute to alignment and safety is also debatable. From an evolutionary perspective, having accurate self-awareness could, paradoxically, make it easier to deceive others—an issue that warrants further examination.

It is to be noted that there are several elements of human metacognition, with specific deficiencies. Human metacognition of own abilities is known to suffer from inaccuracy, especially as regards judgement on general capabilities (Kruger and Dunning, 1999; Dunning, 2011), compared to the correctness of specific responses. Human participants are known to have systemic biases (Kahneman and Tversky, 2013; Glimcher and Rustichini, 2004) away from the rational optimum. These aspects would need to be taken into consideration in the challenge of building a robust and reliable AI, in reference to the overall makeup of human cognition (Müller and Bostrom, 2016).

It is interesting to consider the possibility that metacognitive abilities in LLMs might develop across cognitive domains involving complex perceptual decision-making and theory-of-mind tasks. LLMs have been reported to exhibit task-dependent ToM abilities (Strachan et al., 2024). In humans, there is a known correlation between metacognition and ToM related neural activity (Vaccaro and Fleming, 2018). The relationship between ToM and metacognition in LLMs is not clear, but it would be interesting to analyze and evaluate this particular point. The vulnerability of LLM metacognition might be improved by training. ToM related to social intelligence such as metacognition to others (‘mentalizing’, Frith, 2012) is also significant for AI alignment (Street, 2024). These factors need to be considered before we make a rigorous judgement on the LLMs’ ability to have metacognition.

The methodological limitations of direct prompt questioning of confidence judgement might be augmented by other indirect and behavior-based methods applicable to non-human animals (Hampton, 2009; Templer et al., 2018), e.g., by making the LLMs engage in a betting game where they have an option to avoid betting when the confidence level is low.

In order to address metacognition in LLM properly, we need to have technologically appropriate systems of metaphor. It has been pointed out that there is no appropriate conceptual framework or metaphor for dealing with LLM (Mitchell, 2024), although metaphors such as LLM as stochastic parrots (Bender et al., 2021), collective intelligence (Trott, 2024), an alien (Sejnowski, 2023), and simulators (Shanahan et al., 2023) have been considered. It would be useful to propose conceptual frameworks aligned with rigorous methods for comparing LLMs and humans.

In general, it is difficult to establish a mapping between the behavioral level and the mechanistic level in LLMs (Davies and Khakzar, 2024), because of emergent properties (Wei et al., 2022) and opacity (Mitchell and Krakauer, 2023). In the particular case studied here, it is also difficult to understand the computation of confidence mechanistically because the parametric space of GPT-4 is too large (estimated to be around 1.7 trillion parameters, Sartori and Orrù, 2023). In models with relatively small parameters, such as GPT-2 small, it might be possible to establish a mapping between the mechanistic properties and the behavioral level using mechanistic interpretability (Wang et al., 2022; Liu et al., 2023), which in turn would reveal how metacognition, such as confidence, are computed in LLMs, although there would be concerns that small systems would not be able to exhibit sufficiently high metacognitive abilities. The difficulty of mechanistically interpreting large parameter systems (Sartori and Orrù, 2023) must be somehow overcome.

Apart from temporal variabilities, it remains uncertain whether the findings of this study can be generalized to LLMs other than the specific version of GPT-4 evaluated, such as Anthropic’s Claude, Google’s Gemini, and future versions of GPTs. Parameters such as model architecture, domains of data used for training, context length, multimodal capabilities, and safety guardrails are known to affect the performances of LLMs. Further comparative research is necessary to determine whether the metacognitive deficits observed here reflect characteristics specific to GPT-4’s architecture, or point to more generic fundamental challenges common to current LLM designs based on the Transformer model (Vaswani et al., 2017). It would be interesting to examine how specific configuration and tools used (e.g., Chain-of-thought (CoT), Wei et al., 2023) would affect metacognitive abilities in LLMs, using tests including those employed in this paper.

Finally, within the broader landscape of AI interpretability and emergent behaviors, it is interesting to clarify how shortcomings identified here relate to practical challenges in alignment and safety. Investigations in the metacognitive abilities of AI need to be aligned with corresponding research in humans. Since there is no consensus as to the mechanism of metacognition in humans, with some models suggesting an essential link between metacognition and consciousness, such endeavors cannot be a unidirectional application of cognitive and neurosciences to AI development. Investigation in the metacognitive abilities in AI would hopefully shed light on the mechanism of metacognition in humans, establishing a positive feedback loop between research on AI and human cognition.

In sum, findings reported here would hopefully provide useful measures in the development of metacognitive abilities in the LLMs, considered to be essential in AI alignment and AI safety.

## Data Availability

All datasets used and analyzed in the current study, including the full list of questions and both human and GPT-4 responses, are publicly available at https://github.com/melonsode/metacognition-confidence-gpt. All analysis scripts and code used to generate GPT-4 responses via the OpenAI API are also available at the same repository.
